# *Irpex lacteus* and *Bacillus velezensis* synergistically suppress tomato gray mold by enhancing antioxidant defense and phenylpropanoid metabolism

**DOI:** 10.3389/fpls.2026.1951753

**Published:** 2026-09-28

**Authors:** Kejia Zhang, Ning Guo, Golam Jalal Ahammed, Airong Liu, Shuangchen Chen

**Affiliations:** 1 College of Horticulture and Plant Protection, Henan University of Science and Technology, Luoyang, China; 2 Henan International Joint Laboratory of Stress Resistance Regulation and Safe Production of Protected Vegetables, Luoyang, China; 3 Henan Engineering Technology Research Center for Horticultural Crop safety and Disease Control, Luoyang, China

**Keywords:** antioxidant defense, *Bacillus velezensis*, gray mold, Irpex lacteus, phenylpropanoid metabolism, tomato

## Abstract

Tomato gray mold caused by *Botrytis cinerea* is a devastating disease in protected cultivation, and biological control using beneficial microorganisms offers a sustainable alternative to chemical fungicides. However, the synergistic effects and underlying mechanisms of combining *Irpex lacteus* with *Bacillus velezensis* against tomato gray mold remain unexplored. In this study, we investigated the individual and combined effects of *I. lacteus* and *B. velezensis* on tomato resistance against *B. cinerea*. Both *I. lacteus* and *B. velezensis* effectively suppressed *B. cinerea* growth in dual culture assays.Individual applications of *I. lacteus* fermentation broth and *B. velezensis* cell-free culture filtrate on tomato plants achieved control efficacies of 55.93% and 62.75%, respectively, whereas their combination significantly increased control efficacy to 86.95%. The combined treatment markedly reduced disease severity and pathogen-induced cell death, accompanied by significantly decreased accumulation of malondialdehyde (MDA), hydrogen peroxide (H_2_O_2_), and superoxide anion (O_2_^·^_-_) compared with pathogen-only controls. Furthermore, the combined treatment substantially elevated the activities of antioxidant enzymes (SOD, POD, CAT, APX), phenylpropanoid pathway enzymes (PAL, PPO, CAD, CA-POD, CGA-POD), and increased the contents of total phenolics, total flavonoids, chlorogenic acid, and DPPH radical scavenging capacity. In addition, the combined treatment restored photosynthetic performance, as evidenced by increased maximum quantum efficiency of PSII (Fv/Fm), enhanced activities of Calvin-cycle enzymes (Rubisco and FBPase), and upregulation of photosynthesis-related genes (*FBPA*, *TPI*, *FBPase*, and *SBPase*). These biochemical changes were generally more pronounced in the combined treatment than in single microorganism treatments. Collectively, our results demonstrate that *I. lacteus* and *B. velezensis* synergistically protect tomato against *B. cinerea* by mitigating oxidative stress and activating both antioxidant and phenylpropanoid defense pathways. These findings highlight the potential of combined *I. lacteus* and *B. velezensis* application as a sustainable biocontrol strategy against tomato gray mold.

## 1 Introduction

Gray mold, caused by the necrotrophic fungus *Botrytis cinerea*, ranks among the most destructive diseases affecting tomato (*Solanum lycopersicum* L.) production globally. This pathogen exhibits a remarkably broad host range, infecting over 200 plant species, and is particularly devastating in protected cultivation systems where high humidity and moderate temperatures create optimal conditions for disease development. In tomato, *B. cinerea* primarily attacks senescing or wounded tissues, including leaves, stems, flowers, and mature fruits, causing severe yield losses that can reach 30-50% in severe outbreaks (Wang et al., 2018; Zhao et al., 2022). The postharvest phase is equally concerning, as the pathogen continues to cause fruit decay during storage and transport, resulting in substantial economic losses for growers and the supply chain (Shen et al., 2022). Despite extensive research efforts over the past decades, gray mold remains a persistent threat to tomato production, largely due to the pathogen’s high genetic variability, broad host range, and ability to rapidly evolve resistance to chemical interventions (Williamson et al., 2007).

The management of tomato gray mold has traditionally relied heavily on the frequent application of synthetic fungicides (Soylu et al., 2010). However, this chemical-intensive approach has become increasingly problematic. The emergence of fungicide-resistant *B. cinerea* populations has been documented worldwide, with resistance reported to multiple classes of fungicides, including benzimidazoles, dicarboximides, anilinopyrimidines, and succinate dehydrogenase inhibitors (Leroch et al., 2013; Sudermann et al., 2022). This resistance crisis has forced growers to apply higher doses or more frequent applications, which in turn exacerbates environmental contamination and raises concerns about food safety and human health (Khalid et al., 2021). Additionally, the development of resistant cultivars has been severely hampered by the lack of natural resistance sources in the tomato germplasm and the complex genetic architecture of resistance traits (Ongena and Jacques, 2008). These challenges underscore the urgent need for alternative, sustainable management strategies that reduce dependence on chemical inputs.

When plants are challenged by necrotrophic pathogens such as *B. cinerea*, a rapid accumulation of reactive oxygen species (ROS) occurs at infection sites. This oxidative burst, while serving as a critical signaling event for activating defense responses, can cause membrane lipid peroxidation, protein oxidation, and cellular damage if left unchecked (Mittler et al., 2022). To counteract this oxidative stress, plants have evolved coordinated antioxidant systems. These include enzymatic scavengers such as superoxide dismutase (SOD), peroxidase (POD), catalase (CAT), and ascorbate peroxidase (APX), alongside non-enzymatic antioxidants including phenolic compounds and flavonoids (Mittler et al., 2022; Foyer and Noctor, 2005). Concurrently, the phenylpropanoid pathway contributes to disease resistance by directing carbon flux toward lignin biosynthesis for cell wall reinforcement and the production of phenolic compounds with direct antimicrobial properties (Dixon and Paiva, 1995). Key enzymes in this pathway, including phenylalanine ammonia-lyase (PAL), polyphenol oxidase (PPO), cinnamyl alcohol dehydrogenase (CAD), and peroxidases specific to caffeic acid and chlorogenic acid (CA-POD and CGA-POD), are instrumental in the biosynthesis of lignin, chlorogenic acid, and other defense-related phenolics (Ali et al., 2006; Mitchell et al., 1994). Notably, beneficial microorganisms such as *Bacillus* species and biocontrol fungi are known to prime these defense pathways, enabling plants to mount faster and stronger responses upon subsequent pathogen attack (Shoresh et al., 2010; Pieterse et al., 2014). However, whether co-application of *I. lacteus* and *B. velezensis* synergistically activates these defense pathways in tomato against *B. cinerea* remains unexplored.

Biological control, which harnesses beneficial microorganisms to suppress plant pathogens, has gained considerable momentum as a promising component of integrated disease management (Caulier et al., 2019). Among the diverse array of biocontrol agents, bacteria belonging to the genus *Bacillus* have attracted particular interest due to their robust nature, environmental adaptability, and prolific production of antimicrobial metabolites (Kloepper et al., 2004). *Bacillus velezensis* has emerged as a particularly effective biocontrol bacterium, capable of inhibiting a wide spectrum of fungal phytopathogens through multiple mechanisms. These include the secretion of cyclic lipopeptides (iturins, fengycins, surfactins), polyketides, and volatile organic compounds that directly suppress pathogen growth, as well as the induction of systemic resistance in host plants (Cheffi et al., 2019; Gao et al., 2025). Numerous studies have demonstrated the efficacy of *B. velezensis* against *B. cinerea* in various crops, with mechanisms involving mycelial inhibition, disruption of spore germination, and activation of plant defense responses (Feng et al., 2024). Notably, the cell-free culture filtrate of *B. velezensis* has been shown to retain strong antifungal activity, making it a promising candidate for practical application due to its ease of preparation and consistent bioactivity (Gao et al., 2025).

While bacterial biocontrol agents have been extensively studied, the potential of wood-decaying basidiomycetes in plant disease management remains relatively underexplored. *Irpex lacteus*, a white-rot fungus belonging to the phylum *Basidiomycota*, is well-known for its ability to secrete a suite of extracellular lignin-modifying enzymes, including laccase, manganese peroxidase, and versatile peroxidase (Li et al., 2025). These enzymes enable the fungus to degrade a wide range of recalcitrant organic compounds, including lignin, cellulose, and various xenobiotics (Huang et al., 2026; Li et al., 2025). In the context of biological control, *I. lacteus* has been shown to suppress several soil-borne and foliar pathogens through competition for colonization sites, nutrient depletion, and the secretion of antifungal metabolites (Poveda et al., 2020). Unlike *Bacillus* species, which primarily inhibit pathogens through diffusible antibiotics, *I. lacteus* appears to exert its antagonistic effects mainly through physical overgrowth and nutrient competition, suggesting that the two organisms may employ complementary mechanisms (Gao et al., 2017).

The concept of combining multiple biocontrol agents to achieve enhanced disease suppression has gained increasing attention in recent years. When two or more beneficial microbes with complementary modes of action are co-applied, they may occupy distinct ecological niches, thereby reducing interspecific competition while providing a broader spectrum of antagonistic activities (Contreras-Cornejo et al., 2024). Furthermore, combined microbial treatments can induce more robust and sustained plant immune responses compared with single strains, as different microbes may activate distinct but converging defense signaling pathways (Pieterse et al., 2014). For instance, certain *Bacillus* species are known to trigger induced systemic resistance (ISR) through jasmonic acid and ethylene signaling, while some fungi may activate salicylic acid-dependent pathways, leading to a more comprehensive and durable defense response (Martínez-Terrazas et al., 2026). Despite these theoretical advantages, the specific combination of *I. lacteus* and *B. velezensis* has not been previously evaluated for biocontrol of tomato gray mold. Moreover, the physiological and biochemical mechanisms that might underlie any synergistic interaction between these two phylogenetically distinct microorganisms remain entirely unknown.

In this study, we evaluated the individual and combined effects of *I. lacteus* and *B. velezensis* on tomato resistance against *B. cinerea* by assessing disease severity, plant growth, cell viability, oxidative stress markers, antioxidant enzyme activities, phenylpropanoid pathway metabolism, and accumulation of defense-related secondary metabolites. Our results show that the combined application of the two biocontrol agents significantly alleviated disease severity, promoted plant growth, reduced oxidative damage, enhanced antioxidant enzyme activities, and activated phenylpropanoid metabolism. Furthermore, using the combination of *I. lacteus* and *B. velezensis*, we demonstrated that the synergistic protection against gray mold is associated with coordinated activation of antioxidant defense and phenolic compound accumulation. Overall, this study provides a theoretical basis for developing *I. lacteus*-*B. velezensis* biopreparations as an eco-friendly strategy to control tomato gray mold.

## 2 Material and methods

### 2.1 Plant materials and growth conditions

Tomato (*Solanum lycopersicum* L. cv. ‘Hezuo 903’) seeds were surface-sterilized by soaking in warm water (55 °C) for 30 min and germinated in seedling trays filled with sterilized substrate. Seedlings were grown in a controlled environment chamber at 28/18 °C (day/night) with a 12 h photoperiod and 70-80% relative humidity. At the five- true-leaf stage, seedlings were transplanted into plastic pots and used for treatments.

### 2.2 Pathogen and microbial strains

*Botrytis cinerea* was previously isolated, purified, and maintained in the Laboratory of Stress Biology of Horticultural Crops, Henan University of Science and Technology. This strain could be obtained from General Microorganism Centre of China Microbial Strain Conservation and Management Committee (CGMCC) with the patent number CGMCC No.5547. *B. cinerea* was cultured on potato dextrose agar (PDA) at 28 °C. Spore suspensions were prepared by culturing the fungus in potato dextrose broth (200 mL with 5 mycelial plugs) at 28 °C and 180 rpm for 5 days, and adjusted to 1 × 10^6^ conidia·mL^-1^.*Irpex lacteus* was isolated from tomato leaves and taxonomically classified under CGMCC No.21057. The strain is preserved at the CGMCC. *I. lacteus* was cultured on a selective medium containing lactose 20 g, yeast extract 2 g, MnSO_4_ 0.5 g, agar 20 g, and deionized water 1 L. For liquid fermentation, the fungus was cultured in a medium containing shredded filter paper 10 g, peptone 10 g, KH_2_PO_4–_2 g, MgSO_4_ 0.8 g, deionized water 1 L, pH 5.5-6.0, at 28 °C and 180 rpm for 5 days. The fermentation broth was filtered through sterile gauze to obtain mycelial suspensions (0.9 g dry weight per 100 mL).

*Bacillus velezensis* Mr12 was provided by Professor Zhou from Xinyang Agriculture and Forestry University. *B. velezensis* Mr12 was cultured in Luria-Bertani (LB) broth at 28 °C and 180 rpm for 48 h. The culture was centrifuged at 12,000 rpm for 20 min, and the supernatant was filter-sterilized through 0.22 µm membranes to obtain cell-free culture filtrates.

### 2.3 Experimental design and treatment application

Eight treatments were established: (1) Control (sterile water only), (2) IL (*I. lacteus* only), (3) BV (*B. velezensis* only), (4) IL+BV (*I. lacteus* followed by *B. velezensis* 3 days later), (5) BC (*B. cinerea* only), (6) IL+BC (*I. lacteus* 6 days before *B. cinerea* inoculation), (7) BV+BC (*B. velezensis* 3 days before *B. cinerea* inoculation), and (8) IL+BV+BC (*I. lacteus* and *B. velezensis* 6 and 3 days before *B. cinerea* inoculation, respectively).

For pathogen inoculation, tomato leaves were wounded and sprayed with *B. cinerea* spore suspension (1 × 10^6^ conidia·mL^-1^) on day 0. For *I. lacteus* treatments, 100 mL of fermentation broth containing 0.9 g mycelial dry weight was applied as a root drench at 6 days prior to *B. cinerea* inoculation (day -6). For *B. velezensis* treatments, filter-sterilized culture filtrates were applied as a root drench at 3 days prior to *B. cinerea* inoculation (day -3). For the combined treatment (IL+BV), *B. velezensis* was applied 3 days after *I. lacteus* application (day -6 and day -3, respectively). Each treatment consisted of 20 plants per replicate, with three independent replicates. Samples were collected at 12 days after pathogen inoculation for all physiological and biochemical analyses.

### 2.4 Dual culture assays

For *I. lacteus* versus *B. cinerea*, mycelial plugs (7 mm diameter) of both fungi were placed on opposite sides of PDA plates and incubated at 26 °C. For *B. velezensis* versus *B. cinerea*, a mycelial plug of *B. cinerea* (7 mm diameter) was placed at the center of PDA plates, and sterile filter paper discs (6 mm diameter) loaded with 3 µL of *B. velezensis* culture filtrate were placed at four equidistant points (25 mm from the center). Plates loaded with LB broth served as controls. All plates were incubated at 28 °C, and pathogen colony diameters were measured when the control pathogen reached two-thirds of the plate. Inhibition rates were calculated based on colony radius reduction relative to controls.

### 2.5 Disease assessment and control efficacy

Disease severity was evaluated 12 days after pathogen inoculation using a 0–9 scale. Disease index (DI) was calculated as DI = [Σ (disease grade × number of leaves in that grade)/(total leaves × 9)] × 100. Control efficacy (%) = [(DI of control − DI of treatment)/DI of control] × 100 (Geng et al., 2022). For each treatment, ten leaves were assessed per replicate, and the experiment was repeated three times independently.

### 2.6 Cell death and histochemical staining

For cell viability assessment, tomato leaves were immersed in 0.04% trypan blue solution for 5 h, then destained in 95% ethanol at 65 °C until chlorophyll was removed, and photographed. For microscopic observation, leaf tissues were further cleared with chloral hydrate solution (250 g chloral hydrate dissolved in 200 mL distilled water) to eliminate residual pigments, and then visualized under a stereomicroscope (Li et al., 2025).

### 2.7 Measurement of oxidative stress markers

Malondialdehyde (MDA) content was determined using the thiobarbituric acid (TBA) method as previously described (Kumar and Knowles, 1993). Samples were ground in 5 mL of 0.1% TCA, and the absorbance of the resulting mixture was measured at 532 nm and 600 nm.

Superoxide anion (O_2_^·-^) content was measured following the previous method (Jiang et al., 2021). The supernatant was mixed with phosphate buffer and hydroxylamine hydrochloride, incubated at 25 °C for 1 h, and then combined with p-aminobenzenesulfonic acid and α-naphthylamine solutions. After a 20-min incubation at 25 °C, the mixture was centrifuged at 3,000×g for 3 min, and the absorbance was recorded at 530 nm.

Hydrogen peroxide (H_2_O_2_) content was determined according to the method described in a previous study (Chakrabarty and Datta, 2008). Samples were crushed with 0.1% TCA and subsequently centrifuged at 19,000 ×g for 20 min at 4 °C. The supernatant was combined with 1 mol/L KI solution and K_2_SO_4_ buffer, and absorbance was recorded at 390 nm.

### 2.8 Antioxidant enzyme activities assays

Leaf samples (0.5 g) were homogenized in 5 mL of ice-cold 50 mmol·L^-1^ phosphate buffer (pH 7.8) containing 1 mmol·L^-1^ EDTA. The homogenate was centrifuged at 12,000 g for 20 min at 4 °C, and the supernatant was used for enzyme assays. Catalase (CAT) activity was determined by monitoring the decomposition of H_2_O_2_ at 240 nm (Guo et al., 2024). Superoxide dismutase (SOD) activity was measured by the nitroblue tetrazolium (NBT) photoreduction method (Cakmak and Marschner, 1992). Peroxidase (POD) activity was assayed using guaiacol as substrate, with one unit defined as a 0.01 change in absorbance at 470 nm per minute (Yan et al., 2021). Ascorbate peroxidase (APX) activity was determined according to established protocols (Luo et al., 2025).

### 2.9 Measurement of chlorophyll fluorescence parameters and Calvin-cycle enzyme activities

Chlorophyll fluorescence parameters were measured using a FluorPen FP110 chlorophyll fluorometer (Photon Systems Instruments, Czech Republic). The saturating pulse intensity was set to 30%, and the measuring pulse to 70%. For each treatment, ten representative plants were selected, and the second fully expanded leaf from the apex was dark-adapted for 25 min prior to measurement. The maximum quantum efficiency of PSII photochemistry (Fv/Fm) was then recorded.

Activities of ribulose-1,5-bisphosphate carboxylase/oxygenase (Rubisco) and fructose-1,6-bisphosphatase (FBPase) were determined using enzyme-linked immunosorbent assay (ELISA) kits (Ruixin Biotech, Quanzhou, China) according to the manufacturer’s instructions.

### 2.10 RT-qPCR

Total RNA was extracted from tomato leaves using the FastPure^®^ Universal Plant Total RNA Isolation Kit (Vazyme, Nanjing, China) according to the manufacturer’s instructions. First-strand cDNA was synthesized from 1 µg of total RNA using Takara PrimeScript™ RT regent Kit with gDNA Eraser (Takara, Japan). RT-qPCR was carried out on a CFX96 Real-Time System (Bio-Rad, USA) using TransStart Tip Green qPCR SuperMix (TransGen, Beijing, China). The thermal cycling conditions were as follows: 94 °C for 30 s, followed by 40 cycles of 94 °C for 5 s, 59 °C for 15 s and 72 °C for 10 s. The tomato *Actin* gene was used as the internal reference to normalize gene expression, and relative transcript levels were calculated using the 2_-_^ΔΔCt^ method (Livak and Schmittgen, 2001). The gene-specific primers used for RT-qPCR are listed in **Supplementary Table 2**.

### 2.11 Phenylpropanoid pathway enzyme assays

Phenylalanine ammonia-lyase (PAL) and polyphenol oxidase (PPO) activities were determined according to established protocols (Luo et al., 2025). For PAL activity, leaf tissue was homogenized in 50 mM H_3_BO_3_ buffer (pH 8.8) containing 2% PVPP and 8 mM β-mercaptoethanol. After centrifugation, the supernatant was incubated with L-phenylalanine, and the absorbance was monitored at 290 nm. PPO activity was measured at 398 nm and calculated based on absorbance changes.

Cinnamyl alcohol dehydrogenase (CAD) activity was assayed following the previously described method with minor modifications (Mitchell et al., 1994). Leaf tissue was homogenized in 50 mM Tris-HCl buffer (pH 8.8) containing 5 mM β-mercaptoethanol and 2% PVPP. After centrifugation at 12,000 ×g for 20 min at 4 °C, the supernatant was incubated with 1 mM coniferyl aldehyde and 0.2 mM NADPH in 50 mM Tris-HCl buffer (pH 8.8). The decrease in absorbance at 340 nm was monitored for 5 min.

Caffeic acid peroxidase (CA-POD) and chlorogenic acid peroxidase (CGA-POD) activities were measured as previously described (Ali et al., 2006). Samples were homogenized in 50 mM phosphate buffer (pH 6.0) containing 1% PVPP and 1 mM EDTA. The homogenate was centrifuged at 12,000 ×g for 20 min at 4 °C, and the supernatant was used as the enzyme extract. CA-POD activity was assayed in a reaction mixture containing 50 mM phosphate buffer (pH 6.0), 5 mM caffeic acid, and 5 mM H_2_O_2_. CGA-POD activity was measured using the same reaction mixture with 5 mM chlorogenic acid as the substrate instead of caffeic acid. The increase in absorbance was monitored at 470 nm for CA-POD and at 420 nm for CGA-POD.

### 2.12 Determination of phenolic compounds and antioxidant capacity

Total phenolic content was determined using the Folin-Ciocalteu method with slight modifications based on a previously described protocol (Zhang et al., 2010). Fresh samples were extracted with 20 mL of 80% methanol, and the supernatant was mixed with Folin-Ciocalteu’s reagent for colorimetric analysis. The total phenolic content was calculated based on a standard curve prepared with gallic acid.

Total flavonoid content was measured according to a previously described method with minor modifications (Zheng et al., 2024). Samples were homogenized in methanol/HCl solution, and the absorbance of the extract was measured at 510 nm using a spectrophotometer. The total flavonoid content was calculated based on a standard curve prepared with rutin.

DPPH radical scavenging activity was assessed following previously established protocols (Savatovic et al., 2012). Briefly, 100 µL of sample extract was mixed with 3.9 mL of DPPH solution (0.1 mM in methanol). After incubation in the dark at room temperature for 30 min, the absorbance was recorded at 517 nm.

Chlorogenic acid content was determined by UV-visible spectrophotometry as previously described (Strack et al., 1987). The sample extract was diluted with ethanol, and the absorbance was measured at 327 nm. The chlorogenic acid content was quantified using a standard curve prepared with authentic chlorogenic acid.

### 2.13 Statistical analysis

Three independent biological replicates were performed for all experiments, and the data were presented as mean ± standard deviation (SD). Differences among treatments were assessed by Duncan’s multiple range test at a significance level of *P* < 0.05. To formally evaluate the synergistic interaction between *I. lacteus* (IL) and *B. velezensis* (BV) under *B. cinerea* (BC) stress, two-way ANOVA was conducted on the four BC-inoculated treatments (BC, IL+BC, BV+BC, and IL+BV+BC), with IL and BV as fixed factors. The interaction term (IL × BV) was used to determine whether the combined effect of the two biocontrol agents was non-additive. All statistical analyses were performed using SPSS 21.0 software.

## 3 Results

### 3.1 Antagonistic activities of *I. lacteus* and *B. velezensis* against *B. cinerea*

To investigate whether *I. lacteus* and *B. velezensis* could inhibit the growth of *B. cinerea*, dual culture assays were performed with the two biocontrol agents against the pathogen. The results revealed distinct antagonistic mechanisms of the two biocontrol agents against *B. cinerea*. *B. cinerea* alone grew rapidly and covered almost the entire plate within 8 days with dense mycelia. When co-cultured with *I. lacteus*, the pathogen’s mycelial growth was gradually inhibited. Although *I. lacteus* initially grew slowly without forming a clear inhibition zone, it eventually overgrew the *B. cinerea* colony after contact, with the fungal mycelium becoming denser and growing more rapidly over the pathogen (**Figure 1A**). Quantitative analysis at 8 days after co-cultured showed that *I. lacteus* achieved an inhibition rate of 62.0% (**Figure 1B**). These results indicate that *I. lacteus* has antagonistic activity against *B. cinerea*.

**Figure 1:**
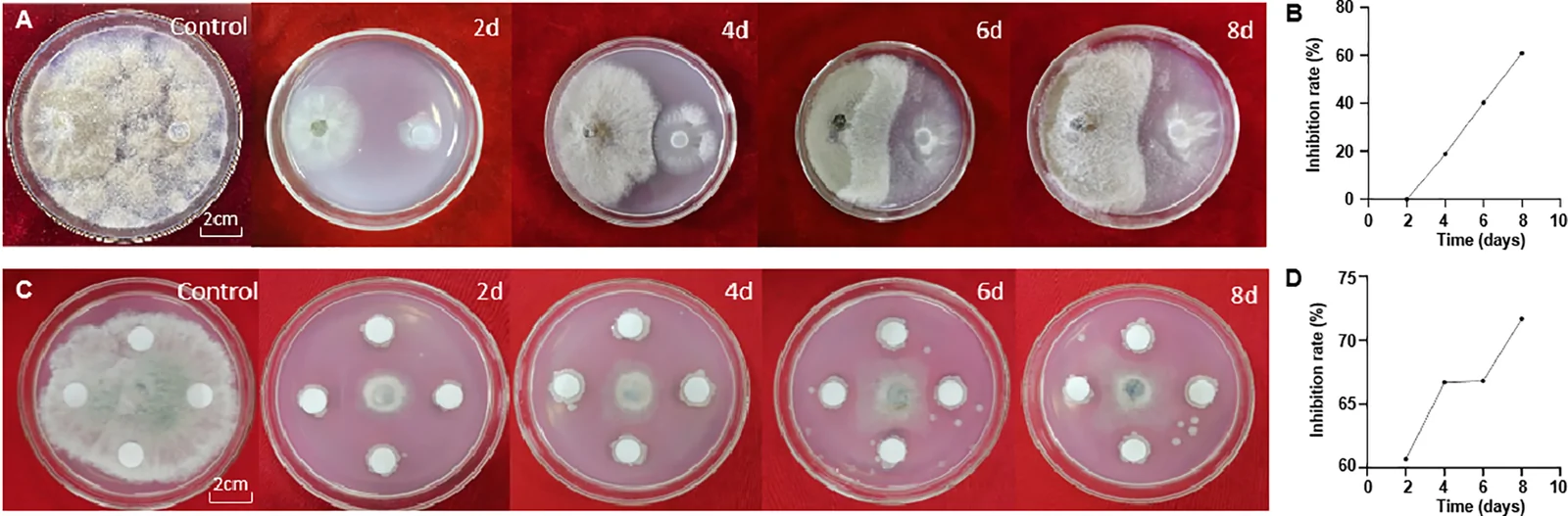
Antagonistic activities of *I. lacteus* and *B. velezensis* against *B. cinerea*. **(A)** Dual culture assay of *I. lacteus* and *B. cinerea* on PDA plates. **(B)** Inhibition rate of *I. lacteus* against *B. cinerea* at different time. **(C)** Dual culture assay of *B. velezensis* and *B. cinerea* on PDA plates. Bar, 2 cm. **(D)** Inhibition rate of *B. velezensis* against *B. cinerea* at different time.

In contrast, *B. velezensis* culture filtrate showed strong inhibitory activity against *B. cinerea* in the disc diffusion assay. The pathogen mycelium could only grow in areas distant from the filter paper discs containing *B. velezensis* filtrate, with a clear inhibition zone forming around the discs. The mycelium that did grow appeared sparse and thin, indicating strong inhibition (**Figure 1C**). The inhibition rate reached approximately 60% at 2 days and increased to 71.5% by 8 days of co-culture (**Figure 1D**). These results indicate that *B. velezensis* culture filtrate has antagonistic activity against *B. cinerea*.

### 3.2 Synergistic effects of *I. lacteus* and *B. velezensis* on plant growth and disease suppression under *B. cinerea* infection

To determine whether *I. lacteus* and *B. velezensis* could enhance tomato resistance against gray mold, we evaluated plant growth, disease severity, and cell death in plants subjected to BC (*B. cinerea* only), IL+BC (*I. lacteus* followed by *B. cinerea*), BV+BC (*B. velezensis* followed by *B. cinerea*), and IL+BV+BC (combined *I. lacteus* and *B. velezensis* followed by *B. cinerea*). At 12 days after pathogen inoculation, tomato plants in the BC treatment exhibited severe disease symptoms, with leaves turning yellow from the bottom upward, wilting, and extensive gray mold covering the leaf surfaces. Plants in the IL+BC and BV+BC treatments showed markedly better growth and milder symptoms with less yellowing and fewer lesions. Notably, the IL+BV+BC treatment displayed the best overall plant vigor, with only minor browning at the leaf tips and almost no visible mold (**Figure 2A**).

**Figure 2:**
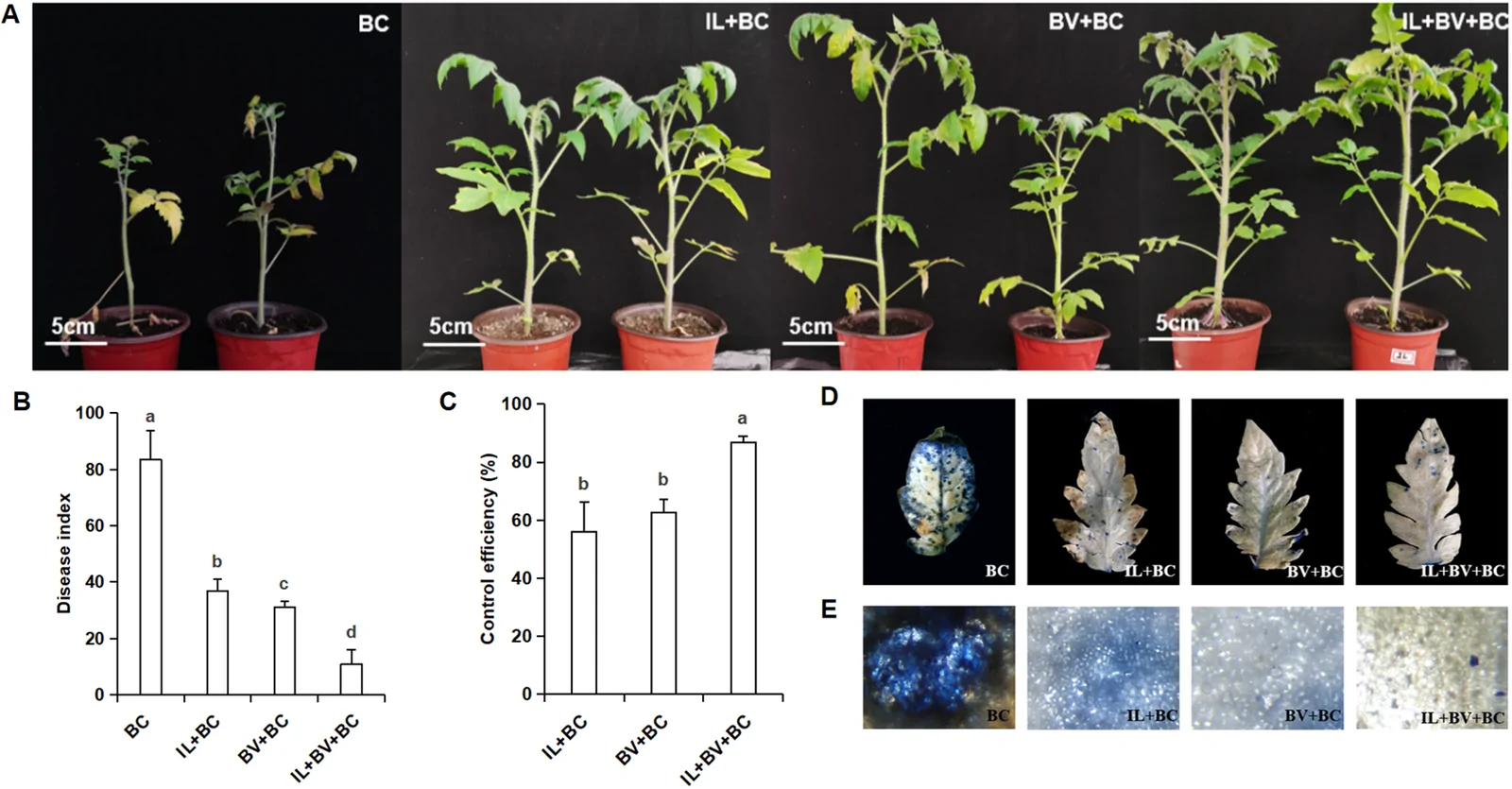
Synergistic effects of *I. lacteus* and *B. velezensis* on tomato gray mold resistance. **(A)** Phenotype of tomato plants under BC, IL+BC, BV+BC, and IL+BV+BC. Bar, 5 cm. **(B)** Disease index of tomato plants under different treatments. **(C)** Control efficacy of *I. lacteus*, *B. velezensis*, and their combination against tomato gray mold. **(D)** Trypan blue staining of tomato leaves showing cell death. **(E)** Higher magnification view of trypan blue staining showing detailed cell death at the cellular level. Data are means ± SD (n = 3). Different letters above bars indicate significant differences according to Duncan’s multiple range test (*P* < 0.05).

Quantitative disease assessment showed the disease index of tomato plants under the BC treatment was 83.5, whereas *I. lacteus* and *B. velezensis* significantly reduced the disease index to 36.8 and 31.1, corresponding to control efficacies of 55.93% and 62.75%, respectively. The combined treatment further reduced the disease index to 10.9, achieving a control efficacy of 86.95%, significantly higher than either single treatment (**Figures 2B, C**). To formally evaluate whether the enhanced effect of the combined treatment reflects a synergistic interaction, we conducted a two-way ANOVA on the four BC-inoculated treatments with IL and BV as fixed factors. The analysis revealed a significant IL × BV interaction for disease index (**Supplementary Table 1**), confirming that the combined effect is synergistic rather than merely additive.

Trypan blue staining was used to assess cell death in tomato leaves. Tomato leaves under BC treatment showed extensive dark blue staining, indicating widespread cell death. IL+BC and BV+BC treatments substantially reduced the staining intensity, while the IL+BV+BC treatment showed only minimal staining, comparable to the control (**Figures 2D, E**). These results demonstrate that pre-application of *I. lacteus* and *B. velezensis*, particularly in combination, effectively protects leaf cells from pathogen-induced death.

### 3.3 Synergistic effects of *I. lacteus* and *B. velezensis* on alleviation of oxidative stress in tomato leaves under *B. cinerea* infection

*B. cinerea* infection caused significant accumulation of oxidative stress markers in tomato leaves. Compared with the control, BC treatment significantly increased MDA, H_2_O_2_, and O_2_^·-^ contents, indicating severe membrane lipid peroxidation and oxidative damage (**Figures 3A–C**). Pre-application of *I. lacteus* or *B. velezensis* significantly reduced these oxidative markers, with the combined treatment showing the greatest reduction. Compared with BC, IL+BC treatment decreased MDA, H_2_O_2_, and O_2_^·-^ contents by 55.40%, 71.50%, and 38.68%, respectively. BV+BC treatment also showed substantial reductions in these oxidative markers. The IL+BV+BC treatment exhibited the lowest levels of all three oxidative stress markers among the inoculated treatments, with values approaching those of the control (**Figures 3A–C**). These results indicate that the biocontrol agents, particularly in combination, effectively alleviate pathogen-induced oxidative stress and membrane damage.

**Figure 3:**
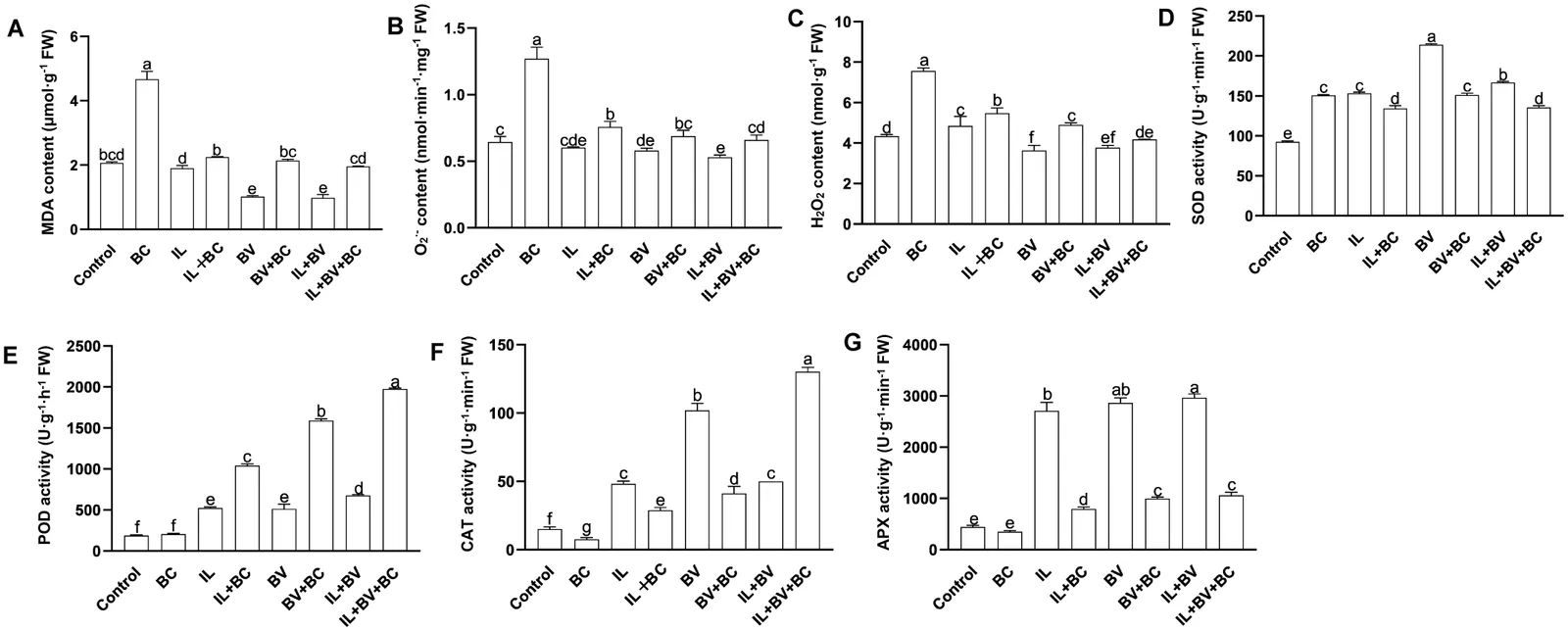
Changes in oxidative damage markers and antioxidant enzyme activities in tomato leaves following biocontrol agent application under *B. cinerea* infection. MDA content **(A)**, O_2_^·-^ content **(B)** H_2_O_2_ content **(C)**, SOD activity **(D)**, POD activity **(E)**, CAT activity **(F)**, and APX activity **(G)** of tomato leaves under Control, IL, BV, IL+BV, BC, IL+BC, BV+BC, and IL+BV+BC treatments. Data are means ± SD (n = 3). Different letters above bars indicate significant differences according to Duncan’s multiple range test (*P* < 0.05).

To evaluate whether the combined application of *I. lacteus* and *B. velezensis* could synergistically enhance the antioxidant capacity of tomato plants, we measured the activities of key ROS-scavenging enzymes, including SOD, POD, CAT, and APX. *B. cinerea* infection significantly suppressed antioxidant enzyme activities in tomato leaves. IL, BV, and IL+BV treatments alone significantly increased SOD, POD, CAT, and APX activities in healthy plants compared with the control (**Figures 3D–G**). Under pathogen stress, IL+BC, BV+BC, and IL+BV+BC treatments significantly elevated SOD, POD, CAT, and APX activities compared with BC alone, with the combined treatment showing the highest levels (**Figures 3D–G**). These findings indicate that pre-application of biocontrol agents primes the antioxidant defense system, enabling more effective ROS scavenging during pathogen challenge, and that the combined treatment provides superior protection through synergistic activation of multiple antioxidant enzymes.

### 3.4 Effects of *I. lacteus* and *B. velezensis* on the photosynthetic performance in tomato leaves under *B. cinerea* infection

*B. cinerea* infection is known to impair photosynthetic efficiency in plants by damaging the photosynthetic apparatus and suppressing carbon assimilation (Sun et al., 2025). To determine whether pre-application of *I. lacteus* and *B. velezensis* could alleviate the photosynthetic inhibition caused by *B. cinerea*, we measured the maximum quantum efficiency of PSII (Fv/Fm) and the activities of two Calvin-cycle enzymes (Rubisco and FBPase). The results showed that *B. cinerea* infection significantly reduced Fv/Fm compared with the Control, indicating damage to PSII photochemical efficiency. Pre-application of *I. lacteus* or *B. velezensis* partially restored Fv/Fm, and the combined treatment (IL+BV+BC) resulted in the highest Fv/Fm among all inoculated treatments (**Figure 4A**). Similarly, the activities of Rubisco and FBPase were markedly decreased by *B. cinerea* infection, whereas IL+BC and BV+BC treatments significantly elevated their activities relative to BC alone. The combined treatment further increased Rubisco and FBPase activities, showing the greatest recovery among the inoculated groups (**Figures 4B, C**).

**Figure 4:**
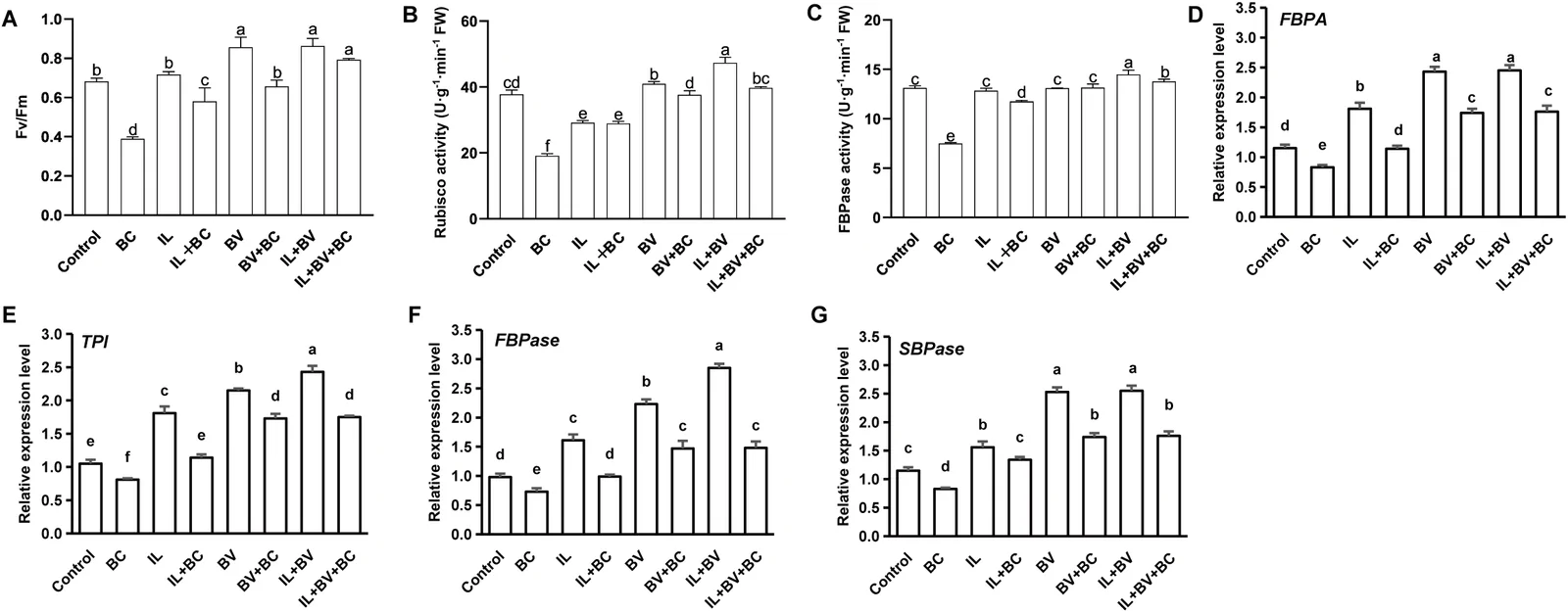
Photosynthetic parameters and Calvin cycle-related gene expression in tomato leaves under different treatments. **(A–C)** Maximum quantum efficiency of PSII (Fv/Fm) **(A)**, Rubisco activity **(B)**, and FBPase activity **(C)** under Control, IL, BV, IL+BV, BC, IL+BC, BV+BC, and IL+BV+BC treatments. **(D–G)** Relative expression levels of *FBPA* **(D)**, *TPI* **(E)**, *FBPase* **(F)** and *SBPase* **(G)** in tomato leaves under Control, IL, BV, IL+BV, BC, IL+BC, BV+BC, and IL+BV+BC treatments. Data are means ± SD (n = 3). Different letters above bars indicate significant differences according to Duncan’s multiple range test (*P* < 0.05).

To further understand the molecular basis of photosynthetic recovery, we examined the transcript levels of four photosynthesis-related genes (*FBPA*, *TPI*, *FBPase*, and *SBPase*) involved in Calvin cycle carbon metabolism. The transcript levels of these genes were relatively low in BC-treated plants compared with the Control. IL+BC and BV+BC partially restored the expression of these genes, whereas the combined treatment (IL+BV+BC) strongly upregulated their expression, with the highest transcript levels observed among all inoculated treatments (**Figures 4D–G**). Collectively, these results demonstrate that pre-application of *I. lacteus* and *B. velezensis*, particularly in combination, effectively alleviates *B. cinerea*-induced photosynthetic impairment by protecting PSII integrity, enhancing Calvin-cycle enzyme activities, and upregulating key photosynthetic genes.

### 3.5 Synergistic effects of *I. lacteus* and *B. velezensis* on phenylpropanoid pathway enzymes, phenolic compounds, and total antioxidant capacity in tomato plants under *B. cinerea* infection

The phenylpropanoid pathway plays a crucial role in plant defense by producing lignin and various phenolic compounds. PAL, PPO, CAD, CA-POD, and CGA-POD activities were significantly elevated by the biocontrol treatments (**Figures 5A–E**). Compared with the Control, IL, BV, and IL+BV treatments substantially increased the activities of PAL, PPO, and CAD in healthy plants. Under pathogen stress, IL+BC, BV+BC, and IL+BV+BC treatments significantly elevated PAL, PPO, and CAD activities compared with BC alone, with the combined treatment showing the highest activities (**Figures 5A–C**). CA-POD and CGA-POD activities, which are involved in the oxidative coupling of hydroxycinnamates during cell wall reinforcement, followed similar patterns. Compared with BC, IL+BC, BV+BC, and IL+BV+BC treatments substantially increased both CA-POD and CGA-POD activities, with the combined treatment exhibiting the greatest enhancement (**Figures 5D, E**). The consistently higher enzyme activities in the combined treatment indicate that *I. lacteus* and *B. velezensis* act synergistically to activate the phenylpropanoid pathway, thereby enhancing the biosynthesis of lignin and defense-related phenolic compounds.

**Figure 5:**
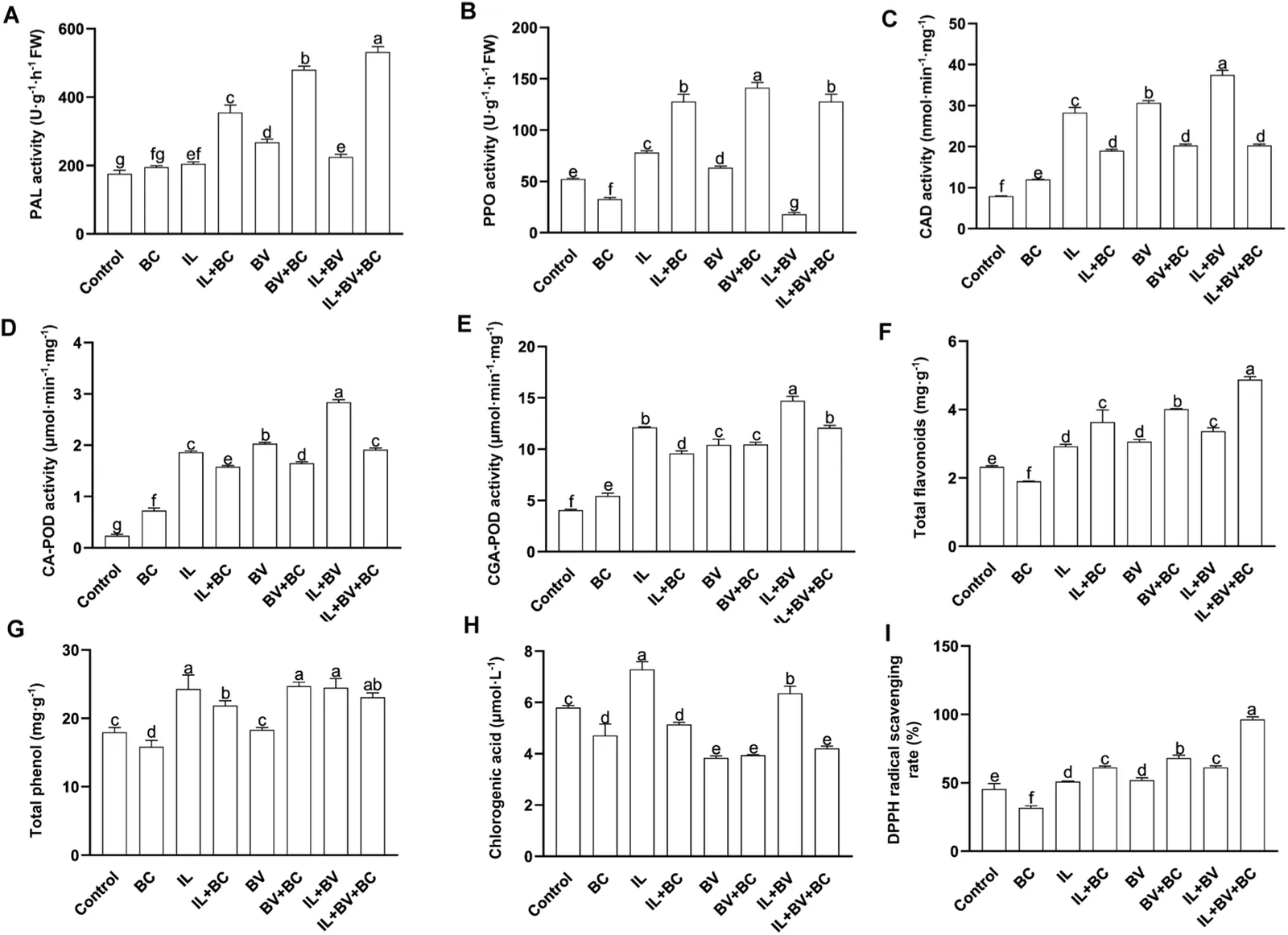
Synergistic effects of *I. lacteus* and *B. velezensis* on phenylpropanoid pathway enzyme activities, phenolic compound accumulation, and antioxidant capacity in tomato leaves under **(B)** cinerea infection. PAL activity **(A)**, PPO activity **(B)**, CAD activity **(C)**, CA-POD activity **(D)**, CGA-POD activity **(E)**, total flavonoid content **(F)**, total phenolic content **(G)**, chlorogenic acid content **(H)**, and DPPH radical scavenging rate **(I)** in tomato leaves under Control, IL, BV, IL+BV, BC, IL+BC, BV+BC, and IL+BV+BC treatments. Data are means ± SD (n = 3). Different letters above bars indicate significant differences according to Duncan’s multiple range test (*P* < 0.05).

In order to determine whether the combined application of *I. lacteus* and *B. velezensis* could synergistically promote the accumulation of defense-related secondary metabolites and enhance the non-enzymatic antioxidant capacity, we measured total flavonoid content, total phenolic content, chlorogenic acid content, and DPPH radical scavenging activity. Total flavonoid and total phenolic contents were significantly increased by *I. lacteus* and *B. velezensis* (**Figures 5F, G**). Under pathogen stress, IL+BC, BV+BC, and IL+BV+BC treatments significantly increased both total flavonoid and total phenolic contents compared with BC alone, with the combined treatment showing the highest accumulation among all inoculated treatments (**Figures 5F, G**) Similarly, chlorogenic acid content and DPPH radical scavenging activity were also enhanced by the biocontrol treatments. Compared with the Control, IL, BV, and IL+BV treatments increased chlorogenic acid content and DPPH scavenging activity (**Figures 5H, I**). Under pathogen stress, IL+BC, BV+BC, and IL+BV+BC treatments showed significantly higher chlorogenic acid content and DPPH scavenging rate compared with BC. The combined treatment IL+BV+BC exhibited the highest chlorogenic acid content and DPPH radical scavenging rate among all inoculated treatments (**Figures 5H, I**), demonstrating that co-application of *I. lacteus* and *B. velezensis* most effectively enhances the non-enzymatic antioxidant capacity and the accumulation of defense-related phenolic compounds.

### 3.6 Effects of *I. lacteus* and *B. velezensis* on expression of defense-related genes in tomato plants under *B. cinerea* infection

To gain molecular insights into the defense mechanisms underlying the synergistic protection conferred by *I. lacteus* and *B. velezensis*, we examined the transcript levels of key genes involved in salicylic acid (SA) signaling (*PR1*), jasmonic acid (JA) biosynthesis and signaling (*LOXD* and *JAR*), JA/ethylene (ET)-dependent defense (*PDF1.2*), and phenylpropanoid metabolism (*PAL5* and *4CL*) in tomato leaves under different treatments (**Figure 6**). *B. cinerea* infection induced the expression of *PR1*, *LOXD*, *JAR*, *PDF1.2*, *PAL5*, and *4CL* compared with the Control, indicating activation of both SA- and JA-mediated defense pathways as well as phenylpropanoid metabolism. Pre-application of *I. lacteus* or *B. velezensis* prior to pathogen challenge (IL+BC and BV+BC) further elevated the transcript levels of these genes compared with BC alone. Notably, the combined treatment (IL+BV+BC) resulted in the highest expression levels of *PR1*, *LOXD*, *JAR*, *PDF1.2*, *PAL5*, and *4CL* among all inoculated treatments (**Figure 6**). These results indicate that the combined application of *I. lacteus* or *B. velezensis* synergistically activates SA- and JA-mediated defense signaling pathways, as well as phenylpropanoid metabolism, at the transcriptional level, providing a molecular basis for the enhanced resistance against *B. cinerea*.

**Figure 6:**
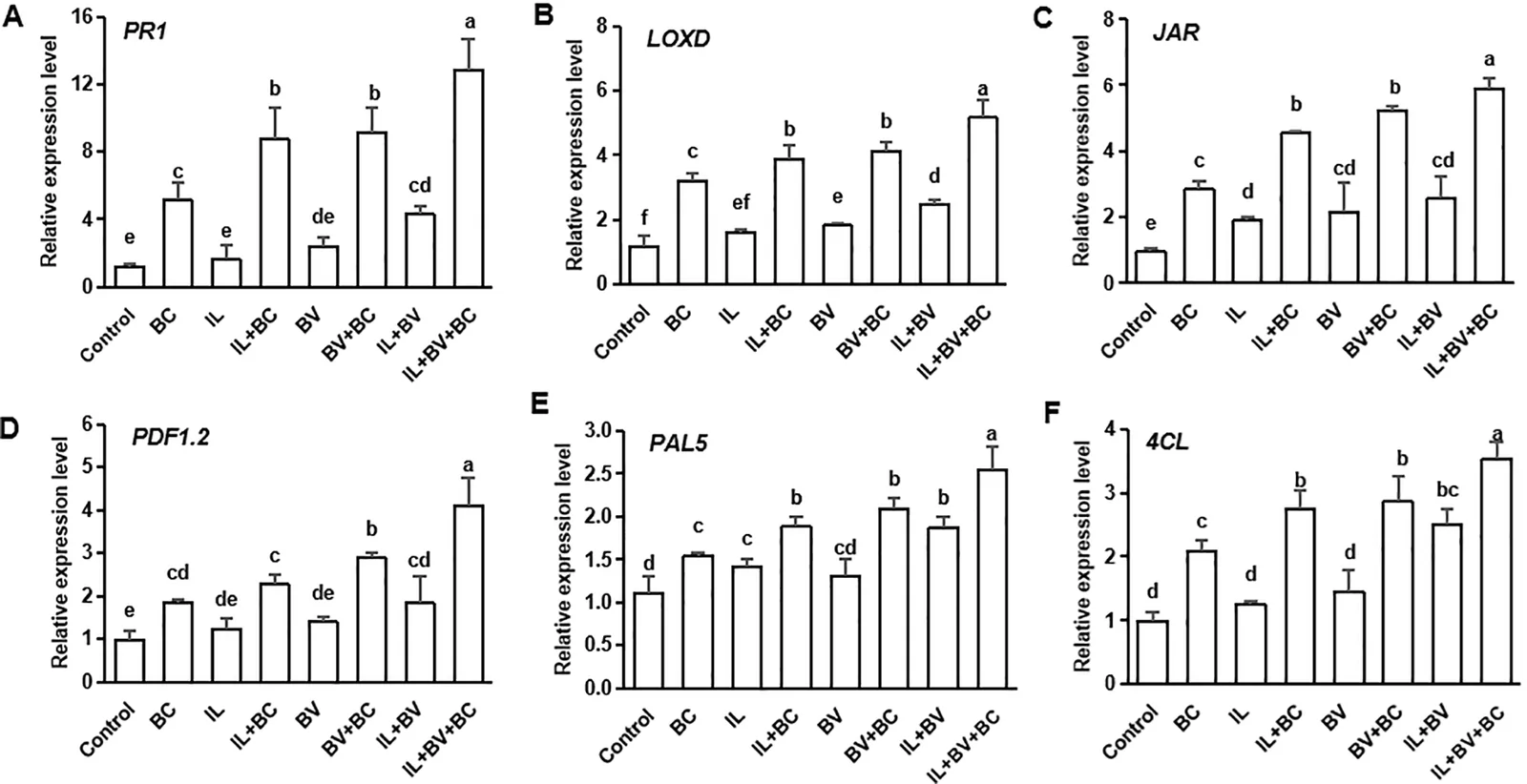
Relative expression level of defense-related genes in tomato leaves under different treatments. Relative mRNA abundance of *PR1* **(A)**, *LOXD* **(B)**, *JAR* **(C)**, *PDF1.2* **(D)**, *PAL5* **(E)**, and *4CL* **(F)** in tomato leaves under Control, IL, BV, IL+BV, BC, IL+BC, BV+BC, and IL+BV+BC treatments. Data are means ± SD (n = 3). Different letters above bars indicate significant differences according to Duncan’s multiple range test (*P* < 0.05).

## 4 Discussion

Several studies have reported the biocontrol potential of *Bacillus velezensis* against tomato gray mold. *B. velezensis* QSE-21 cell-free supernatant has been reported to confer enhanced local and systemic resistance against *B. cinerea* through immune priming and activation of multiple immune signaling pathways (Gao et al., 2025). *Bacillus velezensis* L33a could activate various defense signals and antioxidant pathways in tomatoes, and its genome contains 14 biosynthetic gene clusters predicted to produce antimicrobial compounds (Sun et al., 2024). The endophytic bacterium *Bacillus velezensis* BE1 caused 76.9% inhibition against *B. cinerea* in dual culture and significantly suppressed gray mold incidence and severity in tomato fruit (Aboelez et al., 2024). Meanwhile, recent study has identified *I. lacteus* as a promising biocontrol fungus against *B. cinerea* through volatile organic compounds (VOCs) production (Li et al., 2025). However, the combination of these two phylogenetically distinct microorganisms has not been previously evaluated. Our study provides the first evidence that co-application of a white-rot fungus and a rhizobacterium can achieve synergistic disease suppression through complementary mechanisms (**Figure 2**; **Supplementary Table 1**). Notably, the control efficacy of 86.95% achieved by the combined treatment is comparable to that reported for some chemical fungicides and exceeds the efficacy of many single-strain biocontrol applications (**Figures 2B, C**) (Aboelez et al., 2024; Gao et al., 2025). This suggests that this biological approach could serve as a viable alternative in integrated pest management programs.

The distinct antagonistic patterns observed in dual culture assays, including overgrowth of the pathogen colony by *I. lacteus* and inhibition zone formation by *B. velezensis* culture filtrate, are consistent with previously reported modes of action for these types of microorganisms (**Figure 1**). *I. lacteus* has been shown to suppress fungal pathogens through physical overgrowth and resource competition, while *Bacillus* species are well known for producing diffusible antimicrobial metabolites (Ongena and Jacques, 2008). Recent studies have shown that *I. lacteus* may exert its antagonistic effects through multiple mechanisms, including both physical overgrowth and VOC-mediated inhibition (Li et al., 2025). In contrast, *B. velezensis* culture filtrate produced clear inhibition zones in this study, indicating the presence of diffusible antimicrobial substances. This observation is consistent with the well-documented ability of *B. velezensis* to produce cyclic lipopeptides, including iturins, fengycins, and surfactins, which exhibit strong antifungal activity against *B. cinerea* (Caulier et al., 2019; Ongena and Jacques, 2008). However, since the biocontrol agents were applied to roots while the pathogen was inoculated on leaves, these *in vitro* activities cannot directly explain the disease suppression observed in planta. Instead, the disease suppression observed in planta is most likely attributed to induced systemic resistance (ISR) triggered by root application of the biocontrol agents. This interpretation is supported by the enhanced defense responses observed in leaves, including the activation of antioxidant enzymes, phenylpropanoid metabolism, and upregulation of defense-related genes.

The superior protection conferred by the combined treatment can be attributed to the synergistic activation of multiple layers of antioxidant defense. Pathogen attack typically triggers a rapid burst of reactive oxygen species (ROS), which, if uncontrolled, leads to membrane lipid peroxidation, electrolyte leakage, and programmed cell death (Mittler et al., 2022). In this study, *B. cinerea* infection caused significant accumulation of MDA, H_2_O_2_, and O_2_^·-^, indicating severe oxidative damage (**Figure 3**). Pre-application of *I. lacteus* or *B. velezensis* significantly reduced these oxidative markers, with the combined treatment showing the greatest reduction. This protective effect correlated with enhanced activities of key antioxidant enzymes, including SOD, POD, CAT, and APX (**Figure 4**). The enhanced antioxidant enzyme activities and reduced oxidative damage observed in the combined treatment are typical features of ISR, indicating that the root-applied biocontrol agents prime the plant’s systemic defense responses against the foliar pathogen.

The stronger effect of the combined treatment compared with either single treatment can be explained by complementary pathway activation. The two biocontrol agents may trigger partially overlapping yet distinct defense signaling pathways. *B. velezensis* is known to activate SA-dependent defenses, while fungal elicitors from *I. lacteus* may preferentially engage JA/ET signaling. When applied together, these pathways converge to induce a broader and more robust antioxidant response. This interpretation is supported by gene expression data (**Figure 6**). The coordinated upregulation of these key signaling genes suggests that the two biocontrol agents act synergistically at the transcriptional level to amplify downstream antioxidant defenses, resulting in more effective ROS scavenging and reduced cellular damage during pathogen challenge.

Beyond enhancing antioxidant capacity, the combined treatment also synergistically activated the phenylpropanoid pathway, which is central to plant defense through the production of lignin and antimicrobial phenolic compounds (Dixon and Paiva, 1995). In this study, the combined biocontrol treatment significantly increased the activities of PAL, PPO, CAD, CA-POD, and CGA-POD, which are key enzymes involved in the biosynthesis of lignin, chlorogenic acid, and other defense-related phenolics (**Figure 5**). PAL catalyzes the first committed step of phenylpropanoid metabolism, converting phenylalanine to cinnamic acid (Ali et al., 2006; Dixon and Paiva, 1995). PPO and POD oxidize phenolics to quinones, which are directly toxic to pathogens and serve as precursors for lignin formation (Thipyapong et al., 1995). CAD is involved in the final step of lignin monomer biosynthesis (Mitchell et al., 1994), while CA-POD and CGA-POD participate in the oxidative coupling of hydroxycinnamates during cell wall reinforcement (Ali et al., 2006). The elevated activities of these enzymes likely contributed to the observed accumulation of total phenolics, flavonoids, and chlorogenic acid, as well as enhanced DPPH radical scavenging activity (**Figure 6**).

The stronger effect of the combined treatment can be explained by convergent signaling from SA and JA pathways, which together drive the phenylpropanoid pathway more effectively than either pathway alone. The upregulation of both *PR1* and *JAR* in the combined treatment supports this notion, as these genes represent the two major defense signaling cascades known to regulate phenylpropanoid metabolism (**Figure 6**). Similar findings have been reported for other biocontrol agents. *Trichoderma afroharzianum* TM2–4 was shown to induce antioxidant and phenylpropanoid pathways in tomato, resulting in enhanced immune defense functions (Zhao et al., 2021). The activation of phenylpropanoid pathway enzymes by biocontrol agents has also been documented for *Trichoderma* species (Geng et al., 2022; Shoresh et al., 2010) and *Clonostachys rosea* (Meng et al., 2022). The consistently greater enzyme activities and phenolic compound contents in the combined treatment suggest that *I. lacteus* and *B. velezensis* act synergistically to activate the phenylpropanoid pathway, providing a multilayered chemical defense against *B. cinerea*.

The integrated use of *I. lacteus* and *B. velezensis* offers several practical advantages for sustainable agriculture. Previous studies have shown that combined application of beneficial microbes can provide additive or synergistic biocontrol benefits by enhancing plant defense and restructuring rhizosphere microbial communities (Liu et al., 2026; Song et al., 2015). Arbuscular mycorrhizal fungi combined with *Trichoderma*-enriched bio-organic fertilizer provided additive biocontrol benefits against tomato bacterial wilt by enhancing defense responses and reducing oxidative damage (Song et al., 2015). Similarly, combinations of *Bacillus* species with other biocontrol agents have been reported to improve disease suppression through complementary mechanisms (Liu et al., 2026). The present study extends these findings by demonstrating that a fungal-bacterial combination involving a white-rot fungus can achieve similar synergistic benefits against a foliar pathogen through ISR-mediated defense activation. Furthermore, the use of cell-free culture filtrates offers practical advantages for agricultural application, including simplified preparation, consistent quality control, and reduced regulatory concerns compared with live microbial formulations. The activation of host defense responses, rather than relying solely on direct pathogen inhibition, reduces the selection pressure for resistance development in pathogen populations. These advantages position the *I. lacteus*-*B. velezensis* combination as a promising component of integrated pest management programs for tomato production. Further research under field conditions and exploration of the underlying molecular mechanisms would facilitate the commercial translation of this combined biocontrol strategy.

In summary, this study demonstrates that the combination of *I. lacteus* and *B. velezensis* synergistically protects tomato against *B. cinerea* through root-triggered induced systemic resistance (ISR). Mechanistically, the combined treatment activates both SA and JA/ET defense signaling pathways at the transcriptional level, as evidenced by the upregulation of multiple defense-related genes. This activation leads to two interconnected defense outcomes. The first outcome is the enhanced antioxidant enzyme activities that alleviate oxidative stress and protect cellular integrity. The second outcome is the activation of the phenylpropanoid pathway that drives the accumulation of lignin and antimicrobial phenolic compounds. These two defense layers reinforce each other, creating a robust and sustained defense response that results in reduced disease severity, diminished cell death, and improved plant growth (**Figure 7**). The combined treatment consistently outperformed individual applications, highlighting the potential of dual-microbe biopreparations for sustainable gray mold management. These findings provide a theoretical basis for developing *I. lacteus*-*B. velezensis* biopreparations as an eco-friendly tool to control tomato gray mold and potentially other fungal diseases of horticultural crops.

**Figure 7:**
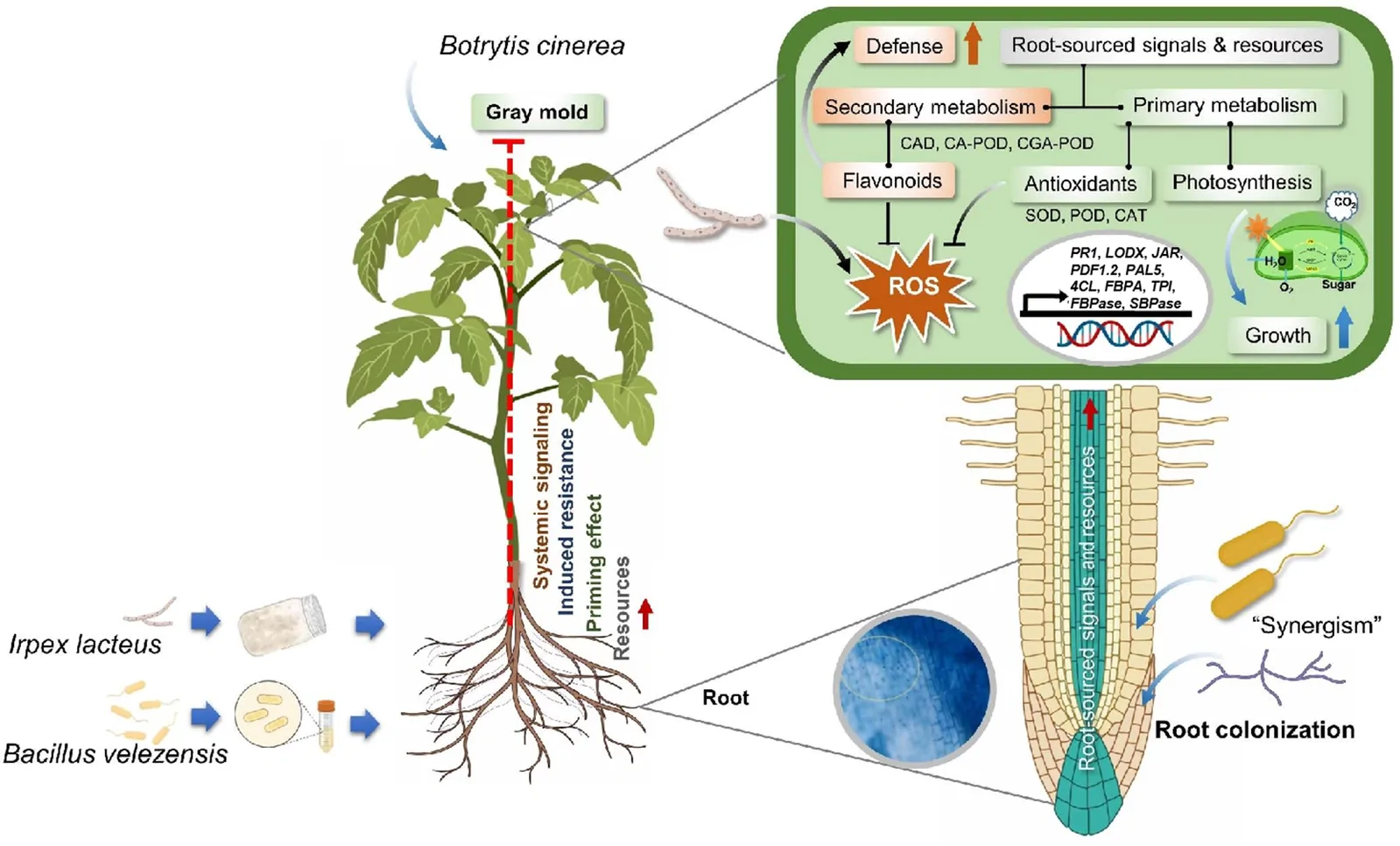
A proposed model illustrating the synergistic mechanisms of *I. lacteus* and *B. velezensis* in inducing tomato resistance against *B. cinerea*. Root application of *I. lacteus* fermentation broth and *B. velezensis* cell-free culture filtrate triggers induced systemic resistance (ISR) in tomato plants. This ISR response involves the activation of SA and JA/ET defense signaling pathways, as evidenced by the upregulation of *PR1*, *LOXD*, *JAR*, and *PDF1.2* at the transcriptional level. The activated signaling pathways coordinately enhance two interconnected defense layers. The first layer is the antioxidant system, including enzymatic components (SOD, POD, CAT, APX) and non-enzymatic components (phenolics, flavonoids), which scavenge ROS and alleviate oxidative damage. The second layer is the phenylpropanoid pathway, driven by key enzymes (PAL, PPO, CAD, CA-POD, CGA-POD), which leads to the accumulation of defense-related phenolic compounds (chlorogenic acid, total phenolics, flavonoids) and lignin biosynthesis for cell wall reinforcement. Concurrently, photosynthetic performance is restored through increased Calvin-cycle enzyme activities (Rubisco, FBPase) and upregulation of photosynthesis-related genes (*FBPA*, *TPI*, *FBPase*, *SBPase*). The synergistic activation of multiple defense levels, including transcriptional, enzymatic, and metabolic levels, ultimately results in enhanced resistance against *B. cinerea*, as reflected by reduced disease severity and improved plant growth.

## Data Availability

The original contributions presented in the study are included in the article/**Supplementary Material**. Further inquiries can be directed to the corresponding author.
